# CYP2B6 polymorphisms and suicidal behaviour in people living with HIV treated with efavirenz-containing combination antiretroviral therapy: a global case–control study

**DOI:** 10.3389/fphar.2026.1734919

**Published:** 2026-04-13

**Authors:** Alejandro Arenas-Pinto, Jacqueline Nordwall, Dianne Carey, Courtney V. Fletcher, Andrew D. Badley, Greg Thompson, Cavan Reilly, Sandra E. Safo, Birgit Grund, Jens Lundgren

**Affiliations:** 1 Medical Research Council Clinical Trials Unit, University College London, London, United Kingdom; 2 Division of Biostatistics and Health Data Science, University of Minnesota, Minneapolis, MN, United States; 3 University of New South Wales, Kirby Institute, Sydney, NSW, Australia; 4 University of Nebraska Medical Center, College of Pharmacy, Omaha, NE, United States; 5 Mayo Clinic, Department on Molecular Medicine, Rochester, MN, United States; 6 Centre of Excellence for Health, Immunity, and Infections, Department of Infectious Diseases, Rigshospitalet, University of Copenhagen, Copenhagen, Denmark

**Keywords:** CYP2B6, efavirenz, HIV, pharmacogenomics, suicidal behaviour, suicidality

## Abstract

**Background:**

We analysed the association between host genetic risk scores (GRSs) predicting efavirenz (EFV) plasma levels and exposure and suicidal behaviours in participants in one of several global antiretroviral therapy (ART) clinical trials linked to the International Network for Strategic Initiatives in Global HIV Trials (INSIGHT).

**Methods:**

We conducted a matched case–control study (two controls per case) on suicide or grade-4 suicidal ideation/attempts or depression. Cases and controls were matched by EFV initiation strategy, study, and follow-up time. CYP2B6-based GRS classified participants as extensive, intermediate, or slow metabolisers. EFV and its metabolites (8-hydroxy-EFV and 7-hydroxy-EFV) were measured in plasma. Conditional logistic regression was used to analyse the associations between GRS categories, EFV levels, and suicidal behaviour.

**Results:**

Among 74 cases and 146 controls, 128 (58.2%) started EFV-containing first-line treatment. Of the treatment-experienced participants, 35% were on EFV at entry. Pre-existing psychiatric conditions and recreational drug-use were more common in cases (16.2%, 25.7%) than in controls (0.7%, 9.6%). On comparing cases to controls, more cases were extensive metabolisers (52% vs. 45%), but there were fewer slow metabolisers (8% vs. 13%). The unadjusted odds ratio (OR) for suicidal behaviour per one more minor allele was 0.76 (95% CI: 0.49–1.18). The OR for being a case vs. a control was 0.57 (95% CI: 0.38–0.86) per doubling of the EFV plasma level.

**Conclusion:**

CYP2B6-based GRS for cytochrome P450 enzymatic activity appeared not to be associated with suicidal behaviour in individuals living with HIV treated with EFV-containing ART combinations. CYP2B6-based GRS effectively predicted EFV exposure, but suicidal behaviour appeared more likely in extensive (normal) metabolisers.

## Introduction

Efavirenz (EFV), a non-nucleoside reverse transcriptase inhibitor (NNRTI), has played a pivotal role in expanding antiretroviral therapy (ART) globally because of its antiviral potency, pharmacokinetic properties, and low cost. More recently, its use has been limited because of its low genetic barrier to resistance and a safety profile that encompasses neuropsychiatric adverse effects, including severe depression and suicidal behaviour (Mollan et al., 2014; Arenas-Pinto et al., 2018). However, EFV remains a suitable treatment option, particularly in people receiving rifampicin-containing treatment for tuberculosis.

Cytochrome P450 2B6 (CYP2B6), a very polymorphic gene, is involved in the metabolism of commonly used drugs, including EFV. Some CYP2B6 alleles such as CYP2B6*9 and CYP2B6*18 have been associated with reduced enzymatic activity, leading to slower metabolism and higher drug exposure (Haas et al., 2004; Holzinger et al., 2012). Similarly, CYP2A6 alleles (e.g., CYP2A6 *4, *9, and *12) have been shown to delay EFV metabolism, particularly in individuals with impaired CYP2B6 function (di Iulio et al., 2009).

Higher plasma concentrations of EFV have been proposed as a mechanism to explain its adverse effects, including liver injury (Yimer et al., 2012) and neuropsychiatric events such as sleep disturbances and dizziness (Marzolini et al., 2001; Clifford et al., 2005), along with ART discontinuation (Cummins et al., 2015). Several host genetic risk scores (GRSs) for increased EFV exposure have been published (Holzinger et al., 2012; Ribaudo et al., 2010; Lubomirov et al., 2011; Leger et al., 2016; Dickinson et al., 2015; Mollan et al., 2017), and recommendations to use a reduced dose in slow metabolisers of CYP2B6 have been published (Desta et al., 2019). A randomised comparison between the standard and reduced EFV doses showed a lower incidence of adverse events in participants receiving the lower dose compared those receiving the standard dose (*p* = 0·008), although the difference was mainly driven by non-psychiatric events (ENCORE1 Study Group, 2014).

We conducted a matched case–control study to explore the association between CYP2B6-based GRS and suicidal behaviour in people living with HIV receiving EFV in four global treatment clinical trials (FIRST, ESPRIT, SMART, and START) linked to the International Network for Strategic Initiatives in Global HIV Trials (INSIGHT).

## Methods

### Study design and participants

Participants were identified from the FIRST, ESPRIT, SMART, and START clinical trials and had consented to genetic analyses. All these studies have been described previously (MacArthur et al., 2006; INSIGHT-ESPRIT Study Group, 2009; Strategies for Management of Antiretroviral Therapy Study Group et al., 2006; INSIGHT START Study Group, 2015); however, we provide a brief description for each. The FIRST (NCT00000922) study randomised treatment-naïve individuals over the age of 13 to start ART with either a protease inhibitor (PI), an NNRTI, or both, in combination with two nucleoside reverse transcriptase inhibitors (NRTIs) between November 1999 and January 2002. The ESPRIT study (NCT00004978) randomised adults on ART between October 1997 and May 2003 to continue their ART with or without interleukin-2. The SMART study (NCT00027352) randomised individuals older than 13 years with a CD4^+^ cell count over 350 cells/mm^3^ to either start or continue their ART without interruption (viral suppression strategy) or to episodic use of ART according to specified CD4^+^ count thresholds (drug conservation strategy) between January 2002 and January 2006. Finally, the START (NCT00867048) study randomised treatment-naïve adults with a CD4^+^ cell count above 500 cells/mm^3^ from April 2009 to December 2013 to either start ART at study entry or to defer treatment initiation until their CD4^+^ cell count declined below 350 cells/mm^3^ or their disease progressed. The studies were approved by the relevant institutional review board or ethics committees at each site or country.

All the studies reported “serious events” unrelated to HIV disease progression during follow-up, irrespective of the exposure to ART. These included deaths and grade-4 adverse events according to the National Institutes of Health (NIH) Division of AIDS toxicity table (U.S. Department of Health and Human Services NIoH, 2017). All reportable events were coded using the Medical Dictionary for Regulatory Activities (MedDRA) by staff blinded to the study arm allocation.

For this case–control study, we considered only participants who were prescribed EFV at trial entry or commenced or switched to EFV during follow-up. Cases of suicidal behaviour were defined as the first occurrence of death by suicide or a grade-4 serious event defined as 1) MedDRA preferred term (PT) of suicide attempt or suicidal ideation, 2) standardised MedDRA query (SMQ) of suicide/self-injury, or 3) MedDRA psychiatric disorders SOC associated with mood disorders. Two controls who had no case-defining events were matched to each case. Matching was based on 1) whether EFV was part of the initial ART after randomisation, was prescribed prior to trial entry, or was commenced after a change in ART during follow-up; 2) study enrolment (FIRST, ESPRIT, SMART, or START); and 3) the follow-up time for the control was at least as long as the time to the event for the case.

### Baseline data

Data on age, sex at birth, region/country of residence, race/ethnicity, likely mode of HIV acquisition, nadir and baseline CD4^+^ cell counts, HIV-RNA, and body mass index (BMI) were collected prior to randomisation. Information regarding pre-existing psychiatric diagnoses (major depression, bipolar disorder, or psychotic disorder, including schizophrenia), history of recreational drug use, and alcohol consumption was collected in the START trial only.

### Genotyping and pharmacokinetic measurements

Participants were genotyped using a custom-content Affymetrix Axiom SNP array, consisting of 770,558 probes, enriched with markers related to immune dysfunction, following a methodology that was previously published (Murray et al., 2023).

Steady-state plasma concentrations of EFV and its main metabolites, namely, 8-hydroxy-EFV (8OH-EFV) and 7-hydroxy-EFV (7OH-EFV), were measured in all the cases and controls in plasma from a single time-point based on the timing of EFV initiation as follows: at baseline for those on EFV prior to randomisation; at least 30 days after initiating EFV at baseline and while still taking EFV; and later during study follow-up. In summary, the assay was modified from our previous work (Sandkovsky et al., 2017), used 20 µL of human plasma processed via liquid–liquid extraction, and was validated (according to FDA guidance) over a range of 20–5,000 ng/mL for EFV and its two metabolites. Analysis was performed using a Shimadzu Nexera integrated UFLC-XR system coupled to an AB Sciex linear-ion trap quadrupole 6500 mass spectrometer (Foster City, CA). The percentage of coefficient of variation (CV) and percentage of deviation were both less than 20% at the lower limit of quantitation (LLOQ) and less than 15% throughout the standard range.

### Genetic risk score for increased exposure to EFV

The primary GRS used in this analysis is a previously validated CYP2B6-based GRS for increased exposure to EFV based on two SNPs, namely, 516G→T (rs3745274) and 983T→C (rs28399499). The primary GRS is based on the combined number of minor alleles for these two SNPs and allows stratifying the participants into three genetic risk categories, namely, extensive metabolisers (0), intermediate metabolisers (1), or slow metabolisers (2) (Ribaudo et al., 2010). Sensitivity analyses were conducted using other published GRSs (Holzinger et al., 2012; Ribaudo et al., 2010; Lubomirov et al., 2011; Leger et al., 2016; Dickinson et al., 2015; Mollan et al., 2017) and limiting the analysis to participants on EFV-containing ART at the time of the event and those starting EFV as first-line ART.

### Statistical analysis

Conditional logistic regression analyses for matched case–control studies were carried out to study the associations of genetic risk categories and EFV concentration (on the log_2_ scale) with suicidal behaviour events. We report unadjusted odds ratios (ORs) and 95% confidence intervals (CIs) for the associations between genetic risk categories and the number of minor alleles, according to the case definition.

The associations between plasma concentrations of EFV and its metabolites 7OH-EFV and 8OH-EFV and the number of minor alleles present were examined using linear regression modelling of EFV measures on the log_2_ scale and the number of minor alleles as a continuous variable. These analyses were restricted to those with a suppressed viral load at the time of specimen collection.

The following sensitivity analyses were performed: 1) adjustment for race/ethnicity, 2) limiting the analysis to cases on an EFV-containing regimen at the time of the event, 3) including only ART-naïve participants, and 4) limiting the analysis to START trial participants and adjusting for pre-existing psychiatric conditions and recreational drug use.

A *p*-value <0.05 was considered significant. Statistical analyses were performed using SAS version 9.4 (SAS Institute, Inc).

## Results

### Characteristics when entering the trial

A total of 220 participants, consisting of 74 cases and 146 controls, were included in this analysis. The characteristics at enrolment are summarised in Table 1; the distributions of age, race–ethnicity, BMI, CD4^+^ cell count, and HIV-RNA were comparable between cases and controls. Most study participants were male (80%), but there was a higher proportion of male participants in the cases (85.1%) than in the controls (76.7%). All 220 participants were treated with EFV-containing ART combinations while participating in their respective studies. Of these, 128 (58.2%) participants were treatment-naïve individuals who started EFV-containing first-line ART, mostly immediately after randomisation. The remaining 92 (41.8%) were ART-experienced participants, 32 of whom were taking EFV at baseline and 60 were switched to EFV during the study period.

**TABLE 1:** Demographic and clinical characteristics of all the study participants.

Characteristics	All (n = 220)	Cases* (n = 74)	Controls (n = 146)
Age in years (median; IQR)	39 (32–45)	37 (29–43)	39 (32–46)
Female sex (%)	45 (20.0)	11 (14.9)	34 (23.3)
Race/Ethnicity (%)			
Black from Africa	16 (7.3)	3 (4.1)	13 (8.9)
Black from outside Africa	39 (17.7)	15 (20.3)	24 (16.4)
Hispanic or from Latin America	53 (24.1)	16 (21.6)	37 (25.3)
Asian+	11 (5.0)	2 (2.7)	9 (6.2)
White, non-Hispanic++	98 (44.5)	36 (48.6)	62 (42.5)
Other	3 (1.4)	2 (2.7)	1 (0.7)
Region of enrolment (%)			
Africa	19 (8.6)	4 (5.4)	15 (10.3)
Asia	10 (4.5)	2 (2.7)	8 (5.5)
Australia	6 (2.7)	3 (4.1)	3 (2.1)
Europe	56 (25.5)	19 (25.7)	37 (25.3)
North America	87 (39.6)	35 (47.3)	52 (35.6)
South America	42 (19.1)	11 (14.9)	31 (21.2)
Trial (%)			
ESPRIT	62 (28.1)	21 (28.4)	41 (28.1)
FIRST	21 (9.6)	7 (9.5)	14 (9.6)
SMART	20 (9.1)	7 (9.5)	13 (8.9)
START	117 (53.2)	39 (52.7)	78 (53.4)
BMI (Kg/m^2^)	24.7 (22.2–27.7)	24.8 (22.5–27.5)	24.6 (22.2–27.7)
Smoking status (%)			
Smoker	66 (30.0)	25 (33.8)	41 (28.1)
Non-smoker	78 (35.4)	23 (31.1)	55 (37.7)
Unknown	76 (34.6)	26 (35.1)	50 (34.2)
IVDU as likely mode of HIV infection (%)	8 (3.6)	3 (4.1)	5 (3.4)
Recreational drug use, ever (%)+++			
Yes	33 (15.0)	19 (25.7)	14 (9.6)
No	84 (38.2)	20 (27.1)	64 (43.8)
Unknown	103 (46.8)	35 (47.3)	68 (46.6)
Heavy alcohol use (%)+++			
Yes	9 (4.1)	5 (6.8)	4 (2.7)
No	102 (46.4)	32 (43.2)	70 (47.9)
Unknown	109 (49.5)	37 (50.0)	72 (49.3)
Past medical history of psychiatric conditions (%) ++++			
Yes	13 (5.9)	12 (16.2)	1 (0.7)
No	104 (47.3)	27 (36.5)	77 (52.7)
Unknown	103 (46.8)	35 (47.3)	68 (46.6)
HIV-RNA at baseline (copies/mL)	8,464 (357–37,430)	6,852 (731–29,071)	10,100 (178–40,442)
HIV-RNA ≤500 copies/mL (%)	27.9	23.0	30.3
CD4 count at baseline (cells/mm^3^)	590 (458–701)	592 (420–726)	587 (478–680)
Nadir CD4 count (cells/mm^3^)	458 (240–571)	458 (250–576)	455 (222–553)
EFV exposure/Use (%)			
Initial ART following randomization or during follow-up	128 (58.2)	43 (58.1)	85 (58.2)
On EFV at baseline	32 (14.5)	11 (14.9)	21 (14.4)
Not ART-naïve at study entry and either started EFV at baseline or switched to EFV during follow-up	60 (27.4)	20 (27.0)	40 (27.4)

*Suicide death or grade-4 mood disorder adverse event.

+All Asians were from Asia, except for one control.

++All white, non-Hispanics were from Australia, Europe, or North America, except for two controls.

+++History of recreational drug use and alcohol use are only available for the START trial.

++++Diagnosis of major depression, bipolar disorder, or psychotic disorder including schizophrenia. Only available for the START trial.

Pre-existing psychiatric conditions (major depression, bipolar disorder, or psychotic disorder) were more frequent in cases than in controls (16.2% and 0.7%, respectively). Recreational drug and heavy alcohol use were also more prevalent in cases (25.7% and 6.8%, respectively) than in controls (9.6% and 2.7%, respectively).

Of the 74 cases, 58 (78.4%) experienced events classified as suicide, suicidal attempt, suicidal ideation, or self-injury. The remaining 16 (21.6%) presented with episodes of depressed mood disorders and disturbances and/or symptoms of grade-4 severity using the DAIDS scale (U.S. Department of Health and Human Services NIoH, 2017). Cases were exposed to EFV-containing ART combinations for a median of 242 [interquartile range (IQR) 68–580] days before the event, but 48.6% had stopped EFV by the time of the event. The median period between EFV discontinuation and the case-defining event was 2.2 years, ranging from 1 day to 5.5 years. Among controls, 19.9% had stopped EFV by the comparable follow-up time of their respective matched case.

### Genetic risk categories

Table 2 shows the distribution of participants by the genetic risk category based on the number of minor alleles present. Most cases were classified as extensive metabolisers (52.1%), while 12.4% of controls and 8.2% of cases were classified as slow metabolisers. A similar distribution was observed among the participants on EFV at the time of the event (Supplementary Table S1). The prevalence of slow metabolisers in the control group was consistent with our main result when calculated using alternative CYP2B6 and CYP2A6 genetic risk scores (Supplementary Table S2). In an unadjusted logistic regression analysis, slower metabolisers showed lower odds of meeting our case definition for suicidal behaviour than extensive metabolisers, although the result was not statistically significant (OR: 0.76; 95% CI: 0.49–1.18 per one more minor allele). The results were consistent when using alternative CYP2B6 and CYP2A6 GRSs (Supplementary Table S2). We observed similar results when the model was adjusted for race/ethnicity (OR: 0.78; 95% CI: 0.50–1.21 per one more minor allele). When the analysis was limited to START trial participants, and the model was adjusted for prior psychiatric conditions, recreational drug use, and excessive alcohol consumption, the OR for being a case per one more minor allele was 0.99 and the 95% CI was 0.41–2.08 (Supplementary Table S3). When suicidal behaviour was defined as “suicide, MedDRA preferred term for suicide attempt or suicidal ideation, or the standardised MedDRA query for suicide/self-injury,” the unadjusted association is nominally significant, and the direction of the association is the same (OR: 0.57; 95% CI 0.33–0.97 per number of minor alleles) (Table 3). When limiting the analysis to participants on EFV-containing ART at the time of the event, the point estimate is similar, and the association is not significant (OR: 0.57; 95% CI: 0.27–1.20 per one more minor allele) (Supplementary Table S3). The results were similar when the analysis was limited to treatment-naïve participants (OR: 0.67; 95% CI: 0.33–1.38 per one more minor allele) (Supplementary Table S4).

**TABLE 2:** Distribution of participants and number (%) based on the genetic risk category (rs3745274 and rs28399499) for the cases and controls.

Genetic risk++	Cases	Controls	Total
Extensive metabolizer	38 (52.1)	65 (44.8)	103 (47.2)
Intermediate metabolizer	29 (39.7)	62 (42.8)	91 (41.7)
Slow metabolizer	6 (8.2)	18 (12.4)	24 (11.0)
Total	73 (100%)	145 (100%)	218 (100%)

+ One case is missing rs28399499, and one control is missing rs3745274.

++ CYP2B6 SNPs; risk determined based on the number of minor alleles:

0, extensive metabolizer

1, intermediate metabolizer

2, slow metabolizer.

**TABLE 3:** Odds ratios per one more minor allele according to the case definition.

Case definition	No. of case/Controls	Odds ratio (OR)+	95% CI	*p*-value
All cases	73/145	0.76	0.49–1.18	0.22
Suicide	12/22	0.45	0.12–1.67	0.24
Suicide or PT for suicide attempt or suicidal ideation	42/84	0.50	0.26–0.99	0.05
Suicide, PT for suicide attempt or suicidal ideation, or SMQ for suicide/self-injury	57/113	0.57	0.33–0.97	0.04

+ OR per 1 allele higher.

Matching considered but no covariates.

### Efavirenz and metabolites levels in peripheral blood

**FIGURE 1:**
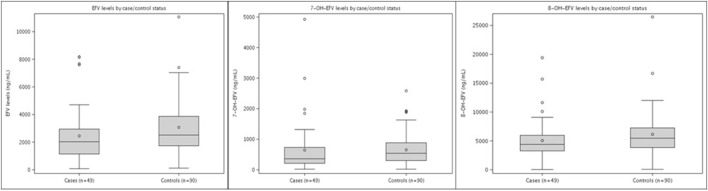
Distribution of EFV markers for the cases and controls (median, IQR).

In samples collected at least 30 days after EFV initiation, the distribution of the plasma concentrations of EFV and its metabolites 7-OH-EFV and 8-OH-EFV was similar in the cases and controls (Figure 1). The median 7-OH-EFV/EFV ratio was 0.21 (IQR 0.15–0.25) in cases and 0.19 (IQR 0.13–0.28) in controls, while the 8-OH-EFV/EFV ratios in the cases and controls were 2.10 (IQR 1.33–2.96) and 2.09 (1.36–3.09), respectively (Supplementary Table S5). Similar results were obtained when the analysis was limited to treatment-naïve participants. Considering the matching criteria but without adjusting for covariates, doubling the EFV concentration in plasma (1 unit increase in log_2_ concentration values) reduces the odds of meeting the case definition (OR: 0.57; 95% CI: 0.38–0.86; *p*: 0.007) (Table 4). Consistent results were obtained when considering only the participants on EFV at the time of the event (OR: 0.60; 95% CI: 0.33–1.09; *p*: 0.09). The plasma levels of EFV and its metabolites were similar in the cases and controls (Supplementary Table S5); however, considering the entire study population (i.e., cases and controls combined), the median concentration of EFV in plasma in slow metabolisers was significantly higher (6,641; IQR: 5,157 ng/mL–8,157 ng/mL) than that in extensive metabolisers (median: 1,794; IQR 1,192 ng/mL–2,479 ng/mL; *p* < 0.001) (Table 5). The median 7-OH-EFV concentration in plasma was higher (median: 1,832; IQR: 1,040 ng/mL–2,406 ng/mL) in slow metabolisers than in extensive metabolisers (median: 333; IQR: 212 ng/mL–540 ng/mL).

**TABLE 4:** Odds ratios of being a case associated with the doubling of EFV concentration.

Case definition	Number of cases/Controls	Odds ratio (OR)+	95% CI	*p*-value
All cases	49/98	0.57	0.38–0.86	0.007
Suicide	6/11	0.32	0.06–1.61	0.16
Suicide or PT for suicide attempt or suicidal ideation	26/48	0.33	0.14–0.79	0.01
Suicide, PT for suicide attempt or suicidal ideation, or SMQ for suicide/self-injury	37/67	0.50	0.30–0.83	0.008

OR per 1 allele higher.

+ Matching considered but no covariates.

**TABLE 5:** Summary of EFV measures based on the number of minor alleles. Cases and controls combined*.

EFV/metabolites	Number of minor alleles present						​
	0		1		2		
	N	Median (IQR)	N	Median (IQR)	N	Median (IQR)	*p*-value**
EFV (ng/mL)	78	1,794 (1,192–2,479)	69	2,807 (2,228–3,818)	19	6,641 (5,157–8,157)	<0.001
7-OH-EFV (ng/mL)	78	333 (212–540)	69	640 (330–864)	19	1832 (1,040–2,406)	<0.001
8-OH-EFV (ng/mL)	78	5,411 (3,808–7,275)	69	5,069 (3,939–6,771)	19	3,432 (3,171–4,437)	0.07
7-OH-EFV/EFV ratio	78	0.20 (0.14–0.26)	69	0.20 (0.15–0.25)	19	0.22 (0.15–0.34)	0.35
8-OH-EFV/EFV ratio	78	2.73 (2.08–3.55)	69	1.61 (1.15–2.10)	19	0.61 (0.42–0.66)	<0.001
Sum of all three measures	78	24,424 (16654–32372)	69	27,313 (21,119–35,550)	19	36,024 (31,314–42,875)	0.022

* Restricted to those with suppressed viral load at the time of specimen collection.

** *p*-value from linear regression modelling EFV measures on log2 scale and the number of minor alleles as the continuous variable.

## Discussion

In this global case–control study, CYP2B6-based GRSs for cytochrome P450 enzymatic activity were not associated with suicidal behaviour in individuals living with HIV who were treated with EFV-containing ART. Our results remained consistent after adjustment for race/ethnicity. In contrast, in a combined analysis of four treatment trials, Mollan et al. (2017) reported an association between CYP2B6 and CYP2A6 polymorphisms that predict higher plasma EFV exposure and suicidal behaviour, but it was only in people of white ancestry. In our analysis, the odds of meeting the case definition decreased as the number of CYP2B6 minor alleles increased, indicating higher odds in extensive metabolisers. This was also observed when the analysis was limited to participants who were ART-naïve at entry and those who had not discontinued EFV before the event.

CYP2B6-based GRS accurately stratified EFV exposure, with slow metabolisers showing higher levels in plasma than extensive metabolisers, while the levels of 8-OH-EFV showed an inverse distribution, all consistent with impaired CYP2B6 activity. However, higher EFV plasma exposure was not associated with suicidal behaviour. Doubling the EFV concentration in plasma reduced the odds of meeting the case definition, even when the analysis was limited to those on EFV at the time of the suicidal event. Similarly, in participants of the ADVANCE trial randomised to EFV-containing ART and concomitantly treated with isoniazid, no association was observed between CYP2B6-GRS and neuropsychiatric events (Taylor et al., 2025). Previous studies have shown that individuals with high EFV plasma levels are at higher risk of CNS toxicities, which primarily include dizziness, sleep disorders, and impaired concentration, but not mood disorders or anxiety (Marzolini et al., 2001; Clifford et al., 2005; Gutierrez et al., 2005). When used at a reduced dose (i.e., 400 mg OD), EFV retained its antiviral efficacy with a better safety and tolerability profile. However, although the overall number of adverse events was lower with the reduced EFV dose compared to that with the standard dose in the ENCORE1 trial, there was no between-group difference in the number of psychiatric adverse events that included depression and suicidality (ENCORE1 Study Group, 2014). Compared to the standard EFV dose (600 mg OD), a reduced dose of 400 mg led to lower EFV but not 8-OH-EFV exposure in the cerebrospinal fluid (CSF) in a sub-study of ENCORE1 (Winston et al., 2015). *In vitro* data show that 8-OH-EFV is particularly toxic to neurons (Tovar-y-Romo et al., 2012; Harjivan et al., 2014), and it has been proposed that extensive EFV metabolisers might be more likely to develop long-term neuropsychiatric toxicities (Decloedt and Maartens, 2013).

EFV has been associated with suicidal behaviour in treatment-naïve individuals in randomised controlled trials, including in those who had stopped EFV before the adverse event (Arenas-Pinto et al., 2018; Mollan et al., 2017). Both the use of recreational drugs and pre-existing psychiatric conditions have been independently associated with suicidal behaviours in people taking EFV; therefore, its use in these population groups is not recommended. In the START trial, the prevalence of pre-existing psychiatric conditions and the use of psychotropic medication were lower in those with pre-specified EFV-containing combinations compared to those with other ART (Arenas-Pinto et al., 2018). However, in the current analysis, we found that the prevalence of pre-existing psychiatric conditions in cases derived from the START study was significantly higher than that in controls. Pre-existing mental health illnesses are known risk factors for suicidal behaviour and severe depressive symptoms in people treated with other drugs, including antimicrobial medications such as cycloserine and dasabuvir (Li et al., 2024).

The main strengths of our analysis are the large number of cases and the inclusion of appropriately matched controls. Although we did not collect data on the social and contextual factors (e.g., poverty, unemployment, and violence exposure) that have been associated with mood disorders and even suicidality, the global nature of the cohort helped to explore the study question in a diverse study population exposed to different socioeconomic and epidemiological backgrounds. However, our analysis included only participants from selected studies (FIRST, ESPRIT, SMART, or START) who had consented to participate in a genomic sub-study, which may have introduced some selection bias. Since EFV was not the randomised intervention in these studies, participants with pre-existing neuropsychiatric conditions might not have been prescribed EFV (Arenas-Pinto et al., 2018). Because information on preexisting conditions, recreational drug use, and excessive alcohol consumption was not routinely collected in all studies, the impact of these might be underestimated in our analysis. We did not collect data on ART adherence in any of the studies. However, virological efficacy was consistently high in all the studies, and, therefore, one could assume that adherence to the EFV-containing combinations was acceptable. Finally, although not all cases were taking EFV at the time of the event, the analysis restricted to those who were showed results that were consistent with the overall findings.

In conclusion, CYP2B6-based GRS for cytochrome P450 enzymatic activity predicts EFV exposure but does appear to predict suicidal behaviour in individuals treated with EFV-containing ART combinations. Suicidal behaviour appeared more frequent among extensive metabolisers (i.e., individuals with normal EFV-metabolising capacity and expected plasma EFV levels) than among slow metabolisers, who have reduced CYP2B6 activity and, consequently, higher EFV exposure. More rapid EFV metabolism may lead to higher 8-OH-EFV-exposure in the CSF, which may explain the trend towards the higher odds of suicidal behaviour events observed in this analysis.

## Data Availability

The data analysed in this study is subject to the following licenses/restrictions: requests for data can be made through the INSIGHT website at http://www.insight-trials.org/index. Proposals are revised by the INSIGHT Scientific Steering Committee. Requests to access these datasets should be directed to http://www.insight-trials.org/index.

## References

[B1] Arenas-Pinto A. Grund B. Sharma S. Martinez E. Cummins N. Fox J. (2018). Risk of suicidal behavior with use of efavirenz: results from the strategic timing of antiretroviral treatment trial. Clin. Infect. Dis. 67 (3), 420–429. 10.1093/cid/ciy051 29538636 PMC6051455

[B2] Clifford D. B. Evans S. Yang Y. Acosta E. P. Goodkin K. Tashima K. (2005). Impact of efavirenz on neuropsychological performance and symptoms in HIV-infected individuals. Ann. Intern Med. 143 (10), 714–721. 10.7326/0003-4819-143-10-200511150-00008 16287792

[B3] Cummins N. W. Neuhaus J. Chu H. Neaton J. Wyen C. Rockstroh J. K. (2015). Investigation of Efavirenz discontinuation in multi-ethnic populations of HIV-positive individuals by genetic analysis. EBioMedicine 2 (7), 706–712. 10.1016/j.ebiom.2015.05.012 26288843 PMC4534686

[B4] Decloedt E. H. Maartens G. (2013). Neuronal toxicity of efavirenz: a systematic review. Expert Opin. Drug Saf. 12 (6), 841–846. 10.1517/14740338.2013.823396 23889591

[B5] Desta Z. Gammal R. S. Gong L. Whirl-Carrillo M. Gaur A. H. Sukasem C. (2019). Clinical pharmacogenetics implementation consortium (CPIC) guideline for CYP2B6 and Efavirenz-Containing antiretroviral therapy. Clin. Pharmacol. Ther. 106 (4), 726–733. 10.1002/cpt.1477 31006110 PMC6739160

[B6] di Iulio J. Fayet A. Arab-Alameddine M. Rotger M. Lubomirov R. Cavassini M. (2009). *In vivo* analysis of efavirenz metabolism in individuals with impaired CYP2A6 function. Pharmacogenet Genomics 19 (4), 300–309. 10.1097/FPC.0b013e328328d577 19238117

[B7] Dickinson L. Amin J. Else L. Boffito M. Egan D. Owen A. (2015). Pharmacokinetic and pharmacodynamic comparison of once-daily efavirenz (400 mg vs. 600 mg) in treatment-naive HIV-infected patients: results of the ENCORE1 study. Clin. Pharmacol. Ther. 98 (4), 406–416. 10.1002/cpt.156 26044067 PMC4744681

[B8] ENCORE1 Study Group (2014). Efficacy of 400 mg efavirenz versus standard 600 mg dose in HIV-infected, antiretroviral-naive adults (ENCORE1): a randomised, double-blind, placebo-controlled, non-inferiority trial. Lancet 383 (9927), 1474–1482. 10.1016/S0140-6736(13)62187-X 24522178

[B11] Gutierrez F. Navarro A. Padilla S. Anton R. Masia M. Borras J. (2005). Prediction of neuropsychiatric adverse events associated with long-term efavirenz therapy, using plasma drug level monitoring. Clin. Infect. Dis. 41 (11), 1648–1653. 10.1086/497835 16267739

[B12] Haas D. W. Ribaudo H. J. Kim R. B. Tierney C. Wilkinson G. R. Gulick R. M. (2004). Pharmacogenetics of efavirenz and central nervous system side effects: an adult AIDS clinical trials group study. AIDS 18 (18), 2391–2400. 15622315

[B13] Harjivan S. G. Wanke R. Ferreira da S. J. L. Marques M. M. Antunes A. M. (2014). The phenolic metabolites of the anti-HIV drug efavirenz: evidence for distinct reactivities upon oxidation with Fremy's salt. Eur. J. Med. Chem. 74, 7–11. 10.1016/j.ejmech.2013.12.022 24440376

[B14] Holzinger E. R. Grady B. Ritchie M. D. Ribaudo H. J. Acosta E. P. Morse G. D. (2012). Genome-wide association study of plasma efavirenz pharmacokinetics in AIDS clinical trials group protocols implicates several CYP2B6 variants. Pharmacogenet Genomics 22 (12), 858–867. 10.1097/FPC.0b013e32835a450b 23080225 PMC3614365

[B9] INSIGHT-ESPRIT Study Group SILCAAT Scientific Committee Abrams D. Levy Y. Losso M. H. Babiker A. (2009). Interleukin-2 therapy in patients with HIV infection. N. Engl. J. Med. 361 (16), 1548–1559. 10.1056/NEJMoa0903175 19828532 PMC2869083

[B10] INSIGHT START Study Group Lundgren J. D. Babiker A. G. Gordin F. Emery S. Grund B. (2015). Initiation of antiretroviral therapy in early asymptomatic HIV infection. N. Engl. J. Med. 373 (9), 795–807. 10.1056/NEJMoa1506816 26192873 PMC4569751

[B15] Leger P. Chirwa S. Turner M. Richardson D. M. Baker P. Leonard M. (2016). Pharmacogenetics of efavirenz discontinuation for reported central nervous system symptoms appears to differ by race. Pharmacogenet Genomics 26 (10), 473–480. 10.1097/FPC.0000000000000238 27509478 PMC5014672

[B16] Li J. Zelmat Y. Storck W. Laforgue E. J. Yrondi A. Balcerac A. (2024). Drug-induced depressive symptoms: an update through the WHO pharmacovigilance database. J. Affect Disord. 350, 452–467. 10.1016/j.jad.2024.01.119 38244800

[B17] Lubomirov R. Colombo S. di Iulio J. Ledergerber B. Martinez R. Cavassini M. (2011). Association of pharmacogenetic markers with premature discontinuation of first-line anti-HIV therapy: an observational cohort study. J. Infect. Dis. 203 (2), 246–257. 10.1093/infdis/jiq043 21288825 PMC3071070

[B18] MacArthur R. D. Novak R. M. Peng G. Chen L. Xiang Y. Hullsiek K. H. (2006). A comparison of three highly active antiretroviral treatment strategies consisting of non-nucleoside reverse transcriptase inhibitors, protease inhibitors, or both in the presence of nucleoside reverse transcriptase inhibitors as initial therapy (CPCRA 058 FIRST study): a long-term randomised trial. Lancet 368 (9553), 2125–2135. 10.1016/S0140-6736(06)69861-9 17174704

[B19] Marzolini C. Telenti A. Decosterd L. A. Greub G. Biollaz J. Buclin T. (2001). Efavirenz plasma levels can predict treatment failure and central nervous system side effects in HIV-1-infected patients. AIDS 15 (1), 71–75. 10.1097/00002030-200101050-00011 11192870

[B20] Mollan K. R. Smurzynski M. Eron J. J. Daar E. S. Campbell T. B. Sax P. E. (2014). Association between efavirenz as initial therapy for HIV-1 infection and increased risk for suicidal ideation or attempted or completed suicide: an analysis of trial data. Ann. Intern Med. 161 (1), 1–10. 10.7326/M14-0293 24979445 PMC4204642

[B21] Mollan K. R. Tierney C. Hellwege J. N. Eron J. J. Hudgens M. G. Gulick R. M. (2017). Race/ethnicity and the pharmacogenetics of reported suicidality with efavirenz among clinical trials participants. J. Infect. Dis. 216 (5), 554–564. 10.1093/infdis/jix248 28931220 PMC5853681

[B22] Murray D. D. Grund B. MacPherson C. R. Ekenberg C. Zucco A. G. Reekie J. (2023). Association between ten-eleven methylcytosine dioxygenase 2 genetic variation and viral load in people with HIV. AIDS 37 (3), 379–387. 10.1097/QAD.0000000000003427 36473831 PMC9894145

[B23] Ribaudo H. J. Liu H. Schwab M. Schaeffeler E. Eichelbaum M. Motsinger-Reif A. A. (2010). Effect of CYP2B6, ABCB1, and CYP3A5 polymorphisms on efavirenz pharmacokinetics and treatment response: an AIDS clinical trials group study. J. Infect. Dis. 202 (5), 717–722. 10.1086/655470 20662624 PMC2919241

[B24] Sandkovsky U. Podany A. T. Fletcher C. V. Owen A. Felton-Coleman A. Winchester L. C. (2017). Impact of efavirenz pharmacokinetics and pharmacogenomics on neuropsychological performance in older HIV-infected patients. J. Antimicrob. Chemother. 72 (1), 200–204. 10.1093/jac/dkw403 27655857 PMC5161051

[B25] Strategies for Management of Antiretroviral Therapy Study Group El-Sadr W. M. Lundgren J. Neaton J. D. Gordin F. Abrams D. (2006). CD4+ count-guided interruption of antiretroviral treatment. N. Engl. J. Med. 355 (22), 2283–2296. 17135583 10.1056/NEJMoa062360

[B26] Taylor J. Maartens G. Sokhela S. Chandiwana N. Akpomiemie G. Venter F. (2025). Pharmacokinetics, pharmacogenetics, and toxicity of co-administered efavirenz and isoniazid. South Afr. J. HIV Med. 26 (1), 1661. 10.4102/sajhivmed.v26i1.1661 40182085 PMC11966721

[B27] Tovar-y-Romo L. B. Bumpus N. N. Pomerantz D. Avery L. B. Sacktor N. McArthur J. C. (2012). Dendritic spine injury induced by the 8-hydroxy metabolite of efavirenz. J. Pharmacol. Exp. Ther. 343 (3), 696–703. 10.1124/jpet.112.195701 22984227 PMC3500535

[B28] U.S. Department of Health and Human Services NIoH (2017). “National institute of allergy and infectious diseases, division of AIDS. Division of AIDS (DAIDS). Division of AIDS (DAIDS) table for grading the severity of adult and pediatric adverse events, corrected version 2.1,” in National institute of allergy and infectious diseases, division of AIDS (Bethesda: Division of AIDS).

[B29] Winston A. Amin J. Clarke A. Else L. Amara A. Owen A. (2015). Cerebrospinal fluid exposure of efavirenz and its major metabolites when dosed at 400 mg and 600 mg once daily: a randomized controlled trial. Clin. Infect. Dis. 60 (7), 1026–1032. 10.1093/cid/ciu976 25501988

[B30] Yimer G. Amogne W. Habtewold A. Makonnen E. Ueda N. Suda A. (2012). High plasma efavirenz level and CYP2B6*6 are associated with efavirenz-based HAART-induced liver injury in the treatment of naive HIV patients from Ethiopia: a prospective cohort study. Pharmacogenomics J. 12 (6), 499–506. 10.1038/tpj.2011.34 21862974

