# Supplementary material for: CYP2B6 polymorphisms and suicidal behaviour in people living with HIV treated with efavirenz-containing combination antiretroviral therapy: a global case–control study
Source: Front Pharmacol. 2026 Apr 13;17:1734919. doi: 10.3389/fphar.2026.1734919 (PMC13111808; doi:10.3389/fphar.2026.1734919)
Supplement: Supplementary file 1 [file Supplementaryfile1.docx]

**Supplementary tables**

**Supplementary table 1. Distribution of Participants^+^ on EFV at the time of the case-defining event, Number (%), by Genetic Risk Category (rs3745274 and rs28399499) for Cases and Controls**

| **Genetic Risk^++^** | **Cases** | **Controls** | **Total** |
| --- | --- | --- | --- |
| Extensive metabolizer | 22 (59.5) | 38 (50.0) | 60 (53.1) |
| Intermediate metabolizer | 11 (29.7) | 30 (39.5) | 41 (36.3) |
| Slow metabolizer | 4 (10.8) | 8 (10.5) | 12 (10.6) |
| Total | 37 (100%) | 76 (100%) | 113 (100%) |

^+^ One case is missing rs28399499.

^++^ CYP2B6 SNPs; risk determined by number of minor alleles:

0 – extensive metabolizer

1 – intermediate metabolizer

2 – slow metabolizer

**Supplementary table 2. Comparison of Odds Ratios (High Risk Slow Metabolizers vs Lower Risk Faster Metabolizers) for Published Genetic Risk Scores**

| **Reference (high risk group)** | **Genes (no. of SNPs) Considered** | **Prevalence of High Risk Group among Controls** | **Odds Ratio (OR)+** | **95% CI** | **P-value** |
| --- | --- | --- | --- | --- | --- |
| Ribaudo et al. (slow) | CYP2B6 (2) | 12.4% | 0.67 | 0.27 – 1.68 | 0.39 |
| Lubomirov et al. (5 or 6) | CYP2B6 (4), CYP2A6 (1), CYP3A4 (1) | 12.6% | 0.71 | 0.28 – 1.79 | 0.46 |
| Leger et al. (slow) | CYP2B6 (3), CYP2A6 (1) | 12.5% | 0.67 | 0.27 – 1.68 | 0.39 |
| Dickinson et al. (slow) | CYP2B6 (2), CYP2A6 (2) | 12.7% | 0.60 | 0.24 – 1.53 | 0.29 |
| Holzinger et al. (8, 9 or 10) | CYP2B6 (3) | 12.5% | 0.67 | 0.27 – 1.68 | 0.39 |
| Mollan et al. (slow) | CYP2B6 (3), CYP2A6 (1) | 12.3% | 0.67 | 0.27-1.68 | 0.39 |

**Supplementary table 3. Odds Ratios Per Number of Minor Alleles According to Case Definition restricted to participants on EFV at the time of the case-defining event**

| **Case Definition** | **No. of Case/Controls** | **Odds Ratio (OR)^+^** | **95% CI** | **P-value** |
| --- | --- | --- | --- | --- |
| All cases | 38/76 | 0.82 | 0. 46 – 1.47 | 0.51 |
| Suicide | 2/4 |  |  |  |
| Suicide or PT for suicide attempt or suicidal ideation | 23/46 | 0.46 | 0.19– 1.13 | 0.09 |
| Suicide, PT for suicide attempt or suicidal ideation, or SMQ for suicide/self-injury | 27/54 | 0.57 | 0.27 – 1.20 | 0.14 |

OR per 1 allele higher.

^+^ Matching considered but no covariates.

**Supplementary table 4. Odds Ratios Per Number of Minor Alleles According to Case Definition restricted to ART naïve participants**

| **Case Definition** | **No. of Case/Controls** | **Odds Ratio (OR)^+^** | **95% CI** | **P-value** |
| --- | --- | --- | --- | --- |
| All cases | 42/85 | 0.84 | 0.46– 1.52 | 0.56 |
| Suicide | 1/2 |  |  |  |
| Suicide or PT for suicide attempt or suicidal ideation | 27/56 | 0.79 | 0.37 – 1.69 | 0.54 |
| Suicide, PT for suicide attempt or suicidal ideation, or SMQ for suicide/self-injury | 33/67 | 0.67 | 0.33 – 1.38 | 0.28 |

OR per 1 allele higher.

^+^ Matching considered but no covariates.

**Supplementary table 5. Median (IQR) EFV Markers for Cases and Controls**

| Marker | **All (n= 139)** | **Cases* (n=49)** | **Controls (n= 90)** |
| --- | --- | --- | --- |
| EFV (ng/mL) | 2415 (1532 – 3434) | 2028 (1144 - 2946) | 2514 (1741 - 3866) |
| 7-OH-EFV (ng/mL) | 517 (264 – 832) | 359 (211 - 731) | 539 (301 - 882) |
| 8-OH-EFV (ng/mL) | 5111 (3688 – 7128) | 4395 (3268 - 5973) | 5464 (3844 - 7244) |
| 7-OH-EFV / EFV ratio | 0.20 (0.14 – 0.28) | 0.21 (0.15 - 0.25) | 0.19 (0.13 - 0.28) |
| 8-OH-EFV / EFV ratio | 2.09 (1.33 – 3.09) | 2.10 (1.33 - 2.96) | 2.09 (1.36 - 3.09) |
| Sum of all 3 measures | 26528 (18995 – 35095) | 21873 (16281 - 30179) | 28827 (21192 - 36024) |
